# Resource acquisition and reproductive strategies of tropical forest in response to the El Niño–Southern Oscillation

**DOI:** 10.1038/s41467-018-03306-9

**Published:** 2018-03-02

**Authors:** Matteo Detto, S. Joseph Wright, Osvaldo Calderón, Helene C. Muller-Landau

**Affiliations:** 1 Department of Ecology and Evolutionary Biology, Princeton University, Guyot Hall, Princeton, NJ 08544-100 USA; 2 Smithsonian Tropical research Institute, Apartado, 0843–03092 Balboa Republic of Panama

## Abstract

The El Niño–Southern Oscillation (ENSO) is the largest source of interannual climate variability in much of the tropics. We hypothesize that tropical plants exhibit interannual variation in reproduction and resource acquisition strategies driven by ENSO that mirrors their seasonal responses. We analyze the relationship of leaf and seed fall to climate variation over 30 years in a seasonally dry tropical forest in Panama where El Niño brings warm, dry, and sunny conditions. Elevated leaf fall precedes the onset of El Niño, and elevated seed production follows, paralleling associations with dry seasons. Our results provide evidence of a shift in allocation from leafing to fruiting in response to a warming phase of ENSO. This shift may enable plants to take advantage of higher light availability, while coping with higher atmospheric water demand and lower water supply. These findings might be an indicator of adaptive strategies to optimize reproduction and resource acquisition.

## Abstract (web summary)

It has been suggested that tree phenology may be regulated by climatic oscillations. Here, Detto et al. present a 30 year tropical forest dataset that suggests leaf and fruit production is coordinated with ENSO cycles, with greater leaf fall observed prior to El Niño followed by greater seed production.

## Introduction

Most tropical plants exhibit distinctive seasonal phenologies driven by the seasonal movements of the Inter Tropical Convergence Zone and associated climate variation, phenologies that are generally interpreted as adaptive^1–4^. For example, in seasonal tropical forests, many tree species flush new leaves during or just prior to the start of the dry season to enable maximal exploitation of the season of high insolation^5,6^. Many plant species in seasonal tropical forests also allocate carbon to fruit production during the dry season and release seeds at the onset of the following wet season^7,8^. This phenological pattern can also be interpreted as adaptive because abundant freshly synthesized sugars are efficiently routed into developing fruits^9^ and optimal conditions are ensured for seed germination, early seedling growth and survival^10^. Selection could only favor these phenological strategies if the associated climatic variation is generally periodic, i.e., events occur with regular frequency. Such periodicity is characteristic not only of seasonal variation, but also of interannual variation, raising the question of whether plants have strategies with respect to longer-term climate modes.

One large scale, periodic pattern that generates interannual variability in climate and plant responses in much of the tropics is the El Niño–Southern Oscillation (ENSO)^11,12^. In general, Central America tends to be dry and warm during the mature phase of an El Niño event, which usually peaks between October and January. In tropical areas where the effect of ENSO is highly prominent, leafing and fruiting can vary with ENSO phases. El Niño events are associated with elevated flower and seed production in Panama^3^, Taiwan^13^, and Borneo^14^. Responses to ENSO have also been detected from satellite observations of NDVI, a proxy for leaf phenology^15^, with differential responses depending on forest type^16^. However, because of difficulties in interpreting remote sensing products in tropical areas, findings on drought responses have been controversial^17,18^, highlighting the need for ground-based studies^19^.

Though interannual variation in seed production and leaf production has been documented in multiple tropical forests, the proximate mechanisms that generate these patterns and the ultimate adaptive values that have favored their evolution remain poorly understood^20^. Proximally, interannual variation in fruit and leaf production in association with climate may reflect variation in available resources and/or shifts in allocation strategies^21^. Under the resource matching hypothesis, plants devote a constant fraction of variable annual resources to reproduction, leaves and roots, resulting in strong, positive temporal correlations among phenophases due entirely to variation in resource availability. Alternatively, plants can shift allocation strategies to optimize fitness under a variable environment, resulting in weaker and possibly negative correlations among phenophases. Differentiating resource matching from shifting allocation is very challenging when a single phenophase is analyzed in isolation, but straightforward if two or more phenophases are analyzed simultaneously.

Ultimately, shifts in allocation would be favored if the relative payoffs of different investments varied predictably among years^21^. For example, we might hypothesize that wind-dispersed species enhance seed production during windy years or that plants prone to embolism reduce leaf area before severe droughts. These and similar hypotheses regarding optimal allocation strategies require that plants are able to anticipate future windy conditions or future drought severity at the time of bud formation and differentiation to avoid subsequent fruit abortion or early termination of leaf life. Such anticipation is feasible if plants respond to a predictable interannual climate cycle; for example, by investing in leaves during the wet phase of a large climatic event, when water is not limiting, and trading leaves for fruits during the following phase of the event when drought is likely.

We hypothesize that the same mechanisms that regulate phenology in relation to seasonal climate variation in tropical forests also operate at the timescales of ENSO. In low-land forests in Central Panama, the focus of our study, leaf fall peaks early in the dry season^22^, with a significant reduction in leaf area index^23^. At the community level, fruiting is strongly seasonal, with a major mode in the second half of the dry season and a smaller mode in the middle of the wet season, driven primarily by understory species^24^. ENSO superimposes alternating wet and dry phases at multi-year intervals on this annual seasonality. Thus, our hypothesis suggests that leaf fall should increase preceding a warm-dry phase and fruiting should increase following a warm-dry phase. For deciduous species, higher litterfall indicates higher standing leaf biomass in the preceding year. However, for evergreen species, higher litterfall could also reflect increased leaf abscission prior to leaf flush to replace old leaves with young leaves thereby increasing photosynthetic efficiency in anticipation of the dry, sunny conditions associated with El Niño events^6,25^.

To explore associations between climatic variability and plant reproduction, observations must be long enough to include the principal modes of climatic variability. A significant ENSO event tends to occur every 2–7 years with quite variable intensity, timing, and duration. Consequently, data sets of less than one decade may contain only sporadic ENSO events, and are of limited use in this context. Longer series would allow analyses of relationships between potential drivers and phenology over a broad range of temporal scales. Correlations between phenological and climatic variability at the frequencies typical of particular climatic cycles would be consistent with causal relationships^26^.

In this study, we use a 30-year data set of fruit and leaf fall to investigate the community level responses of tropical trees and lianas to local climate variability corresponding to the annual seasonal cycle and the modes of ENSO. Specifically, we test the hypothesis that phenological responses to soil moisture, radiation and vapor pressure deficit (VPD) in the frequency typical of ENSO (2–7 years) mirror responses to seasonal cycles, with elevated litterfall prior to a warm-dry phase, and elevated fruiting following a warm-dry phase and with strong interactions between leaf fall and fruit production.

## Results

### Temporal variability of climate and phenology

**Fig. 1:**
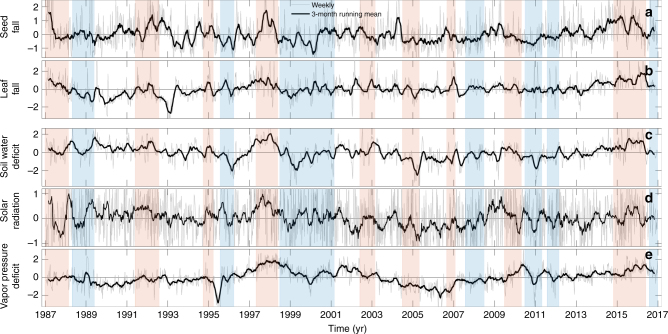
Temporal variation of phenological and climate variables. Weekly series of **a** seed fall (200 traps, 250 species), **b** leaf fall (59 traps, 397 species), and **c**, **d**, **e** climate variables measured in the tropical forest on Barro Colorado Island between 1987 and 2016 in relation to El Niño events (red shading) and La Niña events (blue). All series are seasonally detrended and normalized to zero mean and unit variance to facilitate visual comparison. The dark lines show 3-month running means; the thin gray lines show the weekly time series. *X*-axis ticks indicate January 1st. El Niño and La Niña events are identified based on Oceanic Niño Index values >0.5 °C and less than −0.5 °C, respectively, for at least five consecutive months. Note the major El Niño event in 1997–1998

Climate, seed fall, and leaf fall all varied in association with ENSO (Fig. 1). Leaf and seed fall show similar interannual variability, as also indicated by the power spectra (Supplementary Figure 2). Because of the capacity of soil to store water, temporal variation of soil water deficit is considerably buffered compared with variation of atmospheric processes, and this is reflected in the rapid decline of the power spectrum for soil moisture at small temporal scales. In contrast, radiation, which is directly affected by cloud passage, shows large fine-scale variability, resulting in more equally distributed variance across scales. VPD shows fine-scale variability almost as high as solar radiation, less seasonal variation and larger long time scale variation than soil moisture (Supplementary Figure 2). Interannual variation in soil water deficit and VPD were tightly correlated with the Oceanic Niño Index (Supplementary Figure 3a and b). This correlation was much weaker for solar radiation (Supplementary Figure 3c).

**Fig. 2:**
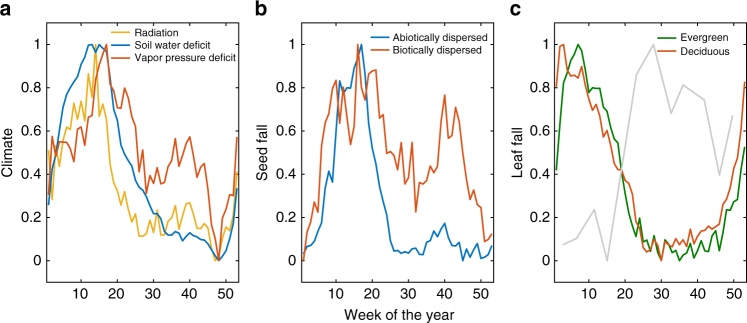
Seasonality of climate and phenological variables. **a** Climate, **b** plant community-level seed fall, **c** leaf fall and leaf area index in the tropical forest on Barro Colorado Island, with plant species separated into groups based on their seed dispersal mode (66 abiotic and 174 abiotic in **b**) and leaf habit (314 evergreen and 63 deciduous in **c**). All variables are normalized to the range of 0–1 to facilitate visual comparison

Strong seasonality is evident in both climate and plant phenology (Fig. 2). Seasonal cycles in solar radiation, soil water deficit and VPD are almost in phase. VPD and, to a lesser extent, solar radiation have a small secondary peak in the wet season (Fig. 2a). Fruiting is bimodal, with a larger first peak centered at the end of the dry season and a smaller second peak in the middle of the wet season (Fig. 2b). Abiotically dispersed species (mostly wind dispersed) release seeds mainly in the windy dry season. The secondary, wet-season peak is driven almost entirely by biotically dispersed species. For both evergreen and deciduous species, leaf fall peaks at the transition from the wet to the dry season (Fig. 2c). The seasonal cycle of leaf fall was inversely related to leaf area index, as shown by the leaf area estimates from the hemispherical photographs (Fig. 2c).

### Coherence and lags between climate and phenology

**Fig. 3:**
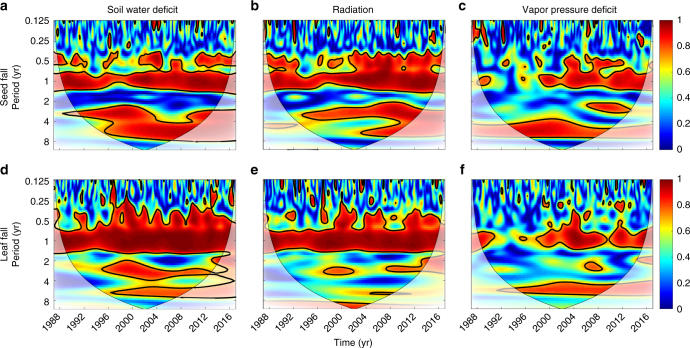
The influence of climate on phenology at multiple temporal scales. Wavelet coherence analysis of seed fall (**a**,** b**, **c**) and leaf fall (**d**, **e**, **f**) with local climate variables indicates strong connections at both seasonal (1 year) and ENSO cycles (2–7 year). Red regions bounded by black contour lines indicate significant coherence (*α* < 0.05). Lighter shading indicates areas outside the cone of influence within which there are sufficient data to reliably test patterns at particular periods and times

There is strong and statistically significant coherence between phenology and environmental drivers both seasonally and at periods of 2–7 years corresponding with ENSO cycles (Fig. 3). At ENSO scales, leaf fall shows the highest coherence with soil water deficit (Fig. 3d) and the lowest with solar radiation (Fig. 3e), with VPD intermediate (Fig. 3f). Seed fall also shows higher coherence with soil water deficit than with solar radiation or VPD at ENSO scales (Fig. 3a–c). At scales >8 years, there is significant coherence between leaf fall and radiation (Fig. 3e). Streamflow discharge, a variable that integrates soil water resources at watershed scale, showed similar coherence patterns as superficial soil water deficit (Supplementary Figure 4).

**Fig. 4:**
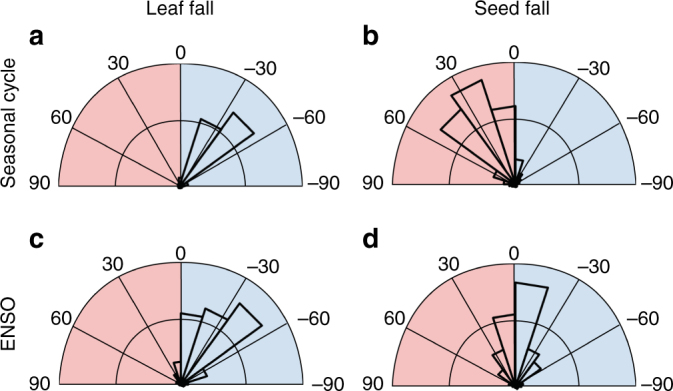
Temporal lags between climate and phenology. Phase-angle histograms for leaf and seed fall with soil water deficit at seasonal cycles (12 months; **a**, **b**) and periods of 2–7 years, corresponding to ENSO cycles (**c**,** d**). Phase angles were calculated for areas inside the cone of influence and for coherence >0.5 in Fig. 3. An angle of 30° corresponds to ~1 month for the seasonal cycle and 2–7 months for the ENSO cycle. Angles in the blue (red) shaded areas indicate that plant phenology leads (lags) climate

At both seasonal and ENSO scales, leaf fall leads the climate variables—that is, peak leaf fall occurs before peak soil water deficit and radiation. This is evident in the analysis of the phase angles the temporal lags between the peaks of the oscillation of two series (Fig. 4 and Supplementary Figure 5). In contrast, seed fall lags the climate variables on seasonal scales, and is more or less in phase with them on ENSO scales. Seasonally, peak seed fall occurs following the peak of the dry season—specifically, about 6 weeks after the peak in radiation and almost 4 weeks after the peak in soil water deficit. For ENSO periods, seed production slightly leads the soil water deficit (−4° ± 36°) and slightly lags solar radiation (4° ± 37°, Supplementary Figure 5), but with an enhanced tendency to lag these climate variables after extreme El Niño events as shown below.

**Fig. 5:**
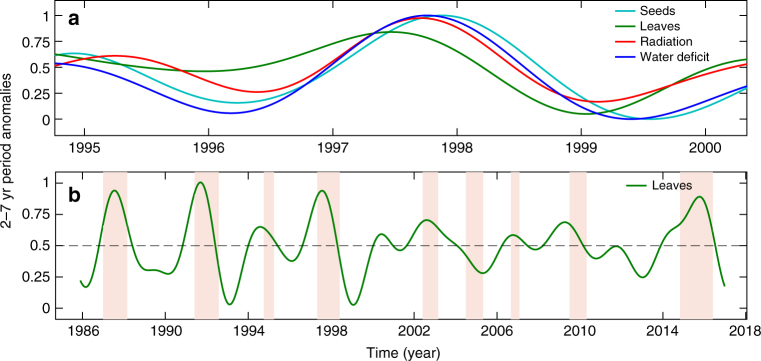
Anomalies are higher during strong ENSO events and lower after these events. **a** Anomalies of solar radiation, soil water deficit, leaf fall, and seed fall around the time of the 1997–1998 El Niño event. Note that the peak of leaf fall preceded peaks of radiation and soil water deficit, while the peak in seeds lagged behind. **b** Anomalies of leaf fall over the entire record show peaks during El Niño events (shaded areas) identified based on Oceanic Niño Index values >0.5 °C for at least five consecutive months. All series are normalized between 0 and 1 to facilitate visual comparison. *X*-axis ticks indicate January 1st

The 1997–1998 El Niño drought was one of the most important events in the 30-year record. It is also an ideal case study because it occurred in the middle of the census interval, so analyses are not biased by edge effects. Both leaf fall and seed production responded positively to the 1997–1998 El Niño drought (Fig. 5a). The peak of leaf fall leads the peak of soil water deficit by 14 weeks (and leads the peak of radiation by 12 weeks). In contrast, the peak of seed fall lags the peak of water deficit by about 4 weeks (and lags radiation by 6 weeks). Unfortunately, the 2015–2016 El Niño cannot be analyzed in similar detail, because the ENSO cycle is not complete and edge effects would distort the smoothed series.

Note that the shape of the oscillation of leaf fall is asymmetrical (green line in Fig. 5a). In particular, the maximum during the ENSO event, in 1997, was greater than the preceding and following maxima, and the minimum that followed the ENSO event, at the beginning of 1999, is much deeper than the minimum preceding the event. The same pattern was observed for other major ENSO events present in the record, 1986–1987, 1991–1992, and 2015–2016 (Fig. 5b). The large peaks in leaf fall at the transition from wet to dry season during ENSO events show that leaf fall was greatest during strong ENSO events. The very low leaf fall in the year following ENSO events raises the possibility that leaf production was also lower during strong ENSO events.

**Fig. 6:**
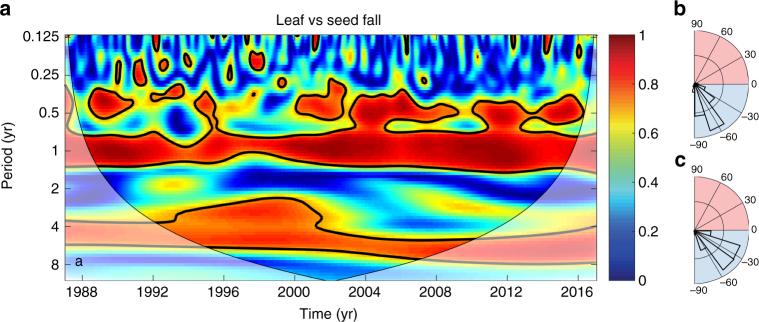
Coordination between leafing and fruiting at multiple temporal scales. **a** Wavelet coherence between leaf fall and seed fall shows strong coordination at both seasonal and ENSO cycles. **b** Phase-angle histogram for leaf fall and seed fall for the seasonal cycle and **c** for periods of 2–7 years, corresponding to the ENSO cycle. Phase angles were calculated for areas inside the cone of influence and for coherence >0.5 in **a**. Negative angles (blue area) indicate that leaf fall leads seed fall

The coherence between seed fall and leaf fall is pervasive across the entire record for both the seasonal and the ENSO cycles (Fig. 6). Community-level seed fall lags leaf fall by ~2–3 months at seasonal scales and by 30°–40° at ENSO scales (corresponding to an average of 5–6 months). This analysis is consistent with their pairwise coherences with climate variables (Figs. 3 and 4).

### LAI and life span

**Fig. 7:**
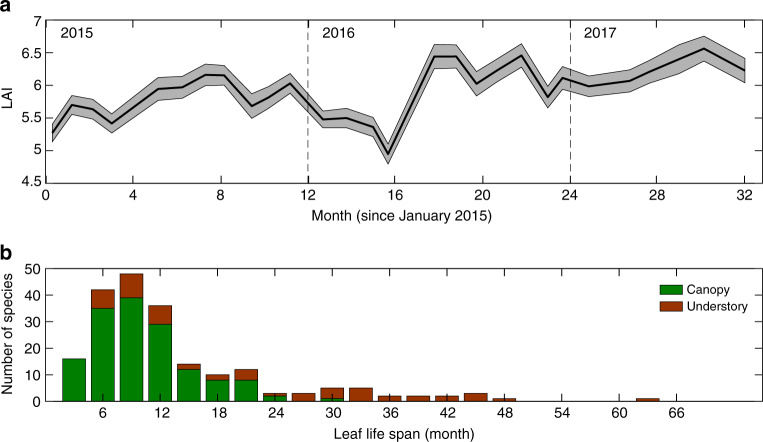
Leaf area index (LAI) was lower during the El Niño 2015–2016 compared to the following period. **a** LAI estimated from hemispherical photographs taken at approximately monthly intervals in 188 locations on Barro Colorado Island. Shaded area includes ± standard errors around the mean. **b** Median leaf life span has a strong mode between 6 and 12 months and is almost always <24 months for canopy species. Median leaf life span is much longer in the understory for both understory shrubs and treelets and saplings of canopy trees. Data were collected in two nearby forests and include 77 species from Parque Metropolitano and 128 species from San Lorenzo

Our measurements of leaf area index (LAI) and leaf life span help to interpret the leaf fall data, which on their own provide only indirect information about temporal variation in leaf production and standing leaf area. LAI was lower during the 2015 El Niño event than during the following neutral period of 2016–2017 (Fig. 7a), consistent with the interpretation that elevated leaf fall during 2015 reduced standing leaf area. Observations of species-specific median leaf life span in two nearby forests indicate a strong modal value between 6 and 12 months, with the vast majority of canopy species having life spans <2 years (Fig. 7b). This suggests that leaf fall provides information mostly on leaf production over the previous 1–2 years.

## Discussion

Phenology, by controlling the rhythms of plants, plays a fundamental role in regulating access to resources, reproduction, competition among species, interactions with consumers and feedbacks to the climate^7,25,27,28^. In tropical forests, phenology is diverse and complex^29,30^ and varies with climatic, edaphic, and biotic factors, such as pollinators and herbivores^31^. Climatic factors are particularly important because they are both proximate and ultimate drivers of plant phenology. Plants often use climate variables as proximate cues for timing phenophases (e.g., to synchronize flowering among conspecifics), and ultimately, climate fluctuations generate variation in resource availability that determines the relative payoffs of different phenological strategies. Due to this intimate link, climate change could significantly alter plant phenology and fitness in the next few decades^32^.

Leaf fall and seed fall responded to both seasonal and interannual climate periodicity in similar ways, consistent with our hypothesis. In this seasonal tropical forest, woody plants drop leaves well before maximum soil moisture deficits associated with the annual dry season (Fig. 2) and with the dry, warm phase of the ENSO cycle (Fig. 5). The lags between peak leaf fall and peak soil water deficits are remarkably similar for seasonal and ENSO cycles (Fig. 4a, c).

These patterns suggest that similar mechanisms underlie plant responses to temporal covariation in water and light availability at seasonal and ENSO timescales. It is an open question whether the consistent interannual patterns of plant phenology documented here reflect adaptive plant strategies. Studies of seasonal patterns of plant phenology infer that leaf fall leads soil water deficits to limit transpiration and drought stress^1^ and that seed fall lags soil water deficits to minimize exposure to seed predators and facilitate germination and seedling growth^24,33^. The documented presence of ENSO activity for at least 130,000 years^34^ suggests that there has been abundant time for selection to act upon plant responses to variability at ENSO scales, lending support to the hypothesis that phenological patterns at these scales may be adaptive. The observed coordination of leaf fall and fruit fall at both seasonal and ENSO scales, also suggests that strategies in which plants switch allocation between leaves and reproduction may be adaptive.

However, the strength of ENSO activity has varied through time as indicated by paleoclimate studies^35^. Even in more recent periods, it is not uncommon to observe several decades of low activity and few events^36^. Alternatively, plant phenological responses at ENSO scales might simply represent side effects of mechanisms that drive adaptive responses at seasonal timescales, side effects that convey little or no significant fitness benefits and may even incur costs. If plants are indeed strategizing with respect to ENSO cycles, this raises the question of how tropical plants track these cycles, i.e., what are the proximate cues that plants are able to sense and utilize.

A limitation of our study is that the leaf fall data sets analyzed here do not provide direct information on temporal variation in leaf production and leaf standing biomass. However, in combination with other data sets, we can infer seasonal and ENSO variation in leaf production, as well as leaf standing biomass. Monthly data collected over two and half years show that stand-level leaf area declines at the end of the wet season coinciding with the peak in leaf fall, and is lower in the dry season and during the 2015–2016 El Niño compared to the following ENSO neutral period (Fig. 7a). Consistent with this, censuses of focal trees and saplings show that leaf flushing has two seasonal peaks on BCI, coinciding with the transitions from wet to dry season and from dry to wet season^37^. We can draw on direct observations of leaf life span (Fig. 7b) to make inferences about ENSO scale variation in leaf production. Leaf life spans suggest that leaf fall is a good proxy for leaf production in the preceding year at interannual scales (for period >2 yr). Because, ENSO periodicity is 4 years on average, our observations of lower leaf fall after major El Niño events (Figs. 5b and 7b) imply lower leaf production during these El Niño events.

In general, seasonal and interannual variation in fruit production have been treated as entirely separate phenomena, yet our results show certain parallels in how stand-level fruit production respond to climate at seasonal and ENSO scales, suggesting that common mechanisms may underlie these responses. At both scales, fruit production is elevated after the peak of solar radiation, a pattern that may reflect in part a direct role of resource availability.

The coordination of leafing and fruiting patterns requires an additional common element that regulates switching allocation. Leaves and fruits compete for terminal buds in many species, and because resources are in limited supply, current investments in one activity result in losses in the investment in the other^38^. A possible explanation for the observed temporal pattern is that the differentiation of meristem to reproductive buds reduces leaf production around El Niño events, while more buds become leaves and fewer buds become reproductive in non-El Niño years. This would generate a cycle consistent with that found here, with the high levels of leaf fall that precede El Niño events representing large allocation to vegetative buds in the year preceding the El Niño event. A shift from vegetative to reproductive growth requires investing large amounts of resources in new reproductive structures, which could in part be supported by retranslocation of foliar nutrients from increased leaf shedding^9^. Manipulative experiments in crops show that removing leaves can counter intuitively increase fruit production^39^.

A reduction in leaf production does not necessarily translate into a period of reduced gross primary productivity. Photosynthesis can be maintained at the same or even higher levels than during the wet La Niña phase of the ENSO cycle because of increased light availability during the relatively dry El Niño phase^40^. Because high levels of atmospheric evaporative demand will increase transpiration, reducing leaf area might be an efficient way to control canopy water loss while maintaining sufficient stomatal conductance to attain high levels of photosynthesis per unit of leaf area and, hence, higher net carbon gain. Furthermore, many fruits are green when unripe, reflecting the presence of chlorophyll. The total photosynthetic area of these green fruits may be a non-trivial contribution to whole-plant photosynthesis while these fruits are developing, often compensating daytime fruit respiratory costs^41^.

Tropical forests contain many different plant species with widely varying resource acquisition and reproductive strategies, which are associated with different phenological responses to seasonal and ENSO cycles. Future research should investigate interspecific variation in phenological responses to ENSO, and seek to identify plant traits associated with such variability. Such research will provide additional insights on the physiological, biochemical, and genetic mechanisms underlying plant phenology and on how future climate change will alter phenological patterns in the tropics.

## Methods

### Study site

Barro Colorado Island (BCI; 9°9′ N, 79°50′ W) is a 15 km^2^ island in Lake Gatun, Panama. Annual rainfall averages 2640 mm, with a distinct dry season between mid-December and mid-April. Mean annual temperature is 26 °C, with minimal seasonal variation^42^. BCI supports tropical moist forest in the Holdridge Life Zone System.

### Meteorological variables

Hydro-meteorological conditions were measured on BCI in the Lutz catchment. Soil water content (swc) was measured gravimetrically every 1–3 weeks for seven soil samples collected at 0–10 cm depth, and interpolated to weekly intervals. A soil water deficit index was calculated as (swc_max_–swc)/(swc_max_–swc_min_), approaching zero for wet conditions and unity for dry conditions. Streamflow was measured for Lutz Creek, which drains the 9.73-ha Lutz catchment, from the water level in a 120° V-notch weir. Solar radiation, air temperature, and relative humidity were measured using a pyranometer (Silicon Pyranometer LI200x, LICOR Bioscience, Lincoln, NE) and a thermistor probe (HMP45AC, Vaisala, Finland), respectively, mounted on the top of a tower extending above the canopy. The tower was originally 42 m tall and was raised to 48 m in 2001 to remain above the surrounding canopy. Measurements were taken at 1-h intervals before 2000 and 15 min intervals thereafter. A supervised QA/QC procedure was applied to eliminate erroneous values and fill gaps using data from other sensors mounted at different heights on the same tower or from a ground station located in a nearby clearing. VPD was computed from temperature and relative humidity in the central part of the day (between 10 AM and 5 PM) when the influence on photosynthesis and stomatal control is largest. Meteorological data were aggregated at weekly time intervals.

### Seed and leaf fall

The rain of fruits and seeds was censused weekly between 5 January 1987 and 31 December 2016 using 200 traps located within a 50-ha plot in the center of BCI^3^. Each trap consisted of a square 0.5-m^2^, open-topped, 1-mm mesh bag supported 0.8–1 m above the ground by a polyvinyl chloride frame. Fruits and seeds were identified to species, counted and further categorized as immature, mature (endosperm filled), or damaged by animals. Our measure of fruit and seed fall for each species in each week was the total number of seed equivalents, henceforth referred to as seeds, calculated as the sum of all mature seeds and mature fruits multiplied by the (species-specific) average number of seeds per fruit.

Leaf litterfall was censused weekly between 18 November 1985 and 31 December 2016 using 59 traps in Poacher’s Peninsula on the south side of BCI^22^. Each trap consisted of a square 0.25-m^2^, open-topped, 1-mm mesh bag supported 0.4 m above the ground by a polyvinyl chloride frame. Leaves were identified to species, oven dried and weighed. Our measure of leaf litterfall for each species in each week was the total oven-dried leaf mass, henceforth referred to as leaves.

Community-level phenologies of seed fall and leaf fall were created by averaging the normalized series of individual woody species of trees, shrubs, and lianas by subtracting the mean and dividing by the standard deviation. Because, we aimed to analyze seasonal, as well as longer-term variation, the series were not seasonally detrended for statistical analyses; however, seasonally detrended series are shown in one figure to illustrate the longer-term patterns. Our seed fall analyses included the 250 species (out of 314 total) that were recorded in >10 censuses, with the exception of *Alseis blackiana*, which was excluded because an unknown portion of its 8-mg seeds pass through the traps and because the methods used to count seeds changed over time. Our leaf fall analyses included the 397 species (out of 527 total) that were recorded in more than three censuses. Results were robust to the choice of threshold for including species (Supplementary Figure 6). The two data sets contained 219 species in common.

### Leaf area index

From January 2015 until April 2017, hemispherical photographs were taken from 188 locations near seed traps with a digital camera (Canon EOS 6D, Canon Inc. Japan) and fisheye lens (Sigma 8 mm f/3.5 EX DG Circular Fisheye Lens, Sigma Corporation of America), approximatively every month during early morning or late afternoon. For each image, a gap fraction was computed as a function of zenith angle by classifying each pixel as sky or vegetation. The leaf area index was estimated using a light penetration model^43^

1 $${\mathrm{LAI}} = - \frac{{\log \left[ {P(\theta )} \right]}}{{\Omega} (\theta)G(\theta)}{\mathrm{cos}}(\theta),$$

where *P* is the gap fraction in the view zenith angle *θ*, *G* is the is the leaf projection function in the direction *θ*, which depends on the leaf inclination angle distribution^44^ and *Ω* is the clumping index and is also a function of *θ*. To minimize problems with distortion, direct radiation at low-sun angle, presence of vertical woody elements and dependence of zenith angle, only a portion of the image directly at the zenith (*θ*| < 4.5°) was used for the calculation of LAI. We used a spherical distribution of leaf inclination *G* = 0.5 and typical clumping index for broadleaf forests *Ω* = 0.9^45^. Although this collection commenced only in 2015, it includes one major El Niño event followed by a neutral period.

### Leaf life span

Leaf life span was measured in two nearby forests. The Bosque Protectora San Lorenzo site (9°17′ N, 79°58′ W) is 30 km NNW of BCI and is relatively wet, with annual rainfall averaging 3200 mm and a 3–4 month long dry season. The Parque Natural Metropolitano site (8°59′ N, 79°33′ W) is 37 km SSE of BCI and is relatively dry, with annual rainfall averaging 1850 mm and a 4–5 month long dry season. In the two forests, we censused all leaves on six branches of understory saplings (or the whole sapling if possible) and all leaves on three randomly chosen, fully sun-exposed branches for canopy trees and lianas every month. Cranes at each site (52 and 42 m tall at the wet and dry sites, respectively) allowed access to the canopy. We used leaves followed from birth to death during 1995–2003 to estimate median leaf longevity for 77 species from Metropolitano and 128 species from San Lorenzo. Total of 114 of these species overlapped with the species collected in the leaf traps on BCI.

### Multiscale analysis

In order to analyze the variability of climate and litterfall at different temporal scales, the series were decomposed using continuous wavelet transforms^46^. The continuous wavelet transform *w*_*n*_(*s*) of a series *x*_*n*_ (*n* = 1,2,…,*N*) observed at regular intervals d*t*, is defined as the convolution of *x*_*n*_ with a scaled and translated wavelet function *f*(*t*)

2 $$w_n(s) = \mathop {\sum}\limits_{i = 1}^N {x_if^ \ast \left( {\frac{{i - n}}{s}{\rm d}t} \right)},$$

where *f*(*t*) = exp(i*k*_0_*t* − *t*^2^/2) is the complex Morlet wavelet with shape parameter *k*_0_ = 6 to ensure the wavelet function satisfies the admissibility condition, * denotes complex conjugate, and *s* is the scale factor corresponding to the period of the oscillation. The local wavelet power spectrum, computed as *s*_*x*,*n*_(*s*) = |*w*_*n*_(s)|^2^ quantifies the variability of *x*_*n*_ at a particular scale. Similarly, given two series *x*_*n*_ and *y*_*n*_, with wavelet transforms *w*_*n*_(*s*) and *h*_*n*_(*s*), the covariance, or wavelet co-spectrum, is given by $c_{xy,n}(s) = w_n(s)h_n^ \ast (s)$. Because, the series have finite length, errors occur near the edges, so the spectra and co-spectra are evaluated only inside a cone of influence where edge effects are negligible. The cone tapers for larger scales, as the length of the series becomes comparable with the period of the oscillations, reducing the time interval where statistical significance can be tested.

The degree of coupling (strength and phase shift) between series was quantified with wavelet coherence analysis^47^. In brief, wavelet coherence measures the normalized covariance between two time series in a manner similar to a coefficient of determination (or *R*^2^) as

3 $$wc_n(s) = \frac{{\left| {\left\langle {c_{xy,n}(s)} \right\rangle } \right|^2}}{{\left\langle {s_{x,n}^{}(s)} \right\rangle \left\langle {s_{y,n}^{}(s)} \right\rangle }},$$

where 〈.〉 indicates smoothing in both time and scale^36^. Like *R*^2^, *wc*_*n*_(*s*) takes values between zero—indicating perfectly uncorrelated series—and one indicating perfectly correlated series. The degree of correlation is independent of the time shift between the series.

The time shift or phase-angle *a*_*n*_(*s*) between two series was estimated as the arctangent of the ratio between the real and imaginary part of *c*_*xy,n*_(*s*).

4 $$a_n(s) = {\mathrm{tan}}^{ - 1}\left( {\frac{{\Im \{ c_{xy,n}^{}(s)\} }}{{\Re \{ c_{xy,n}^{}(s)\} }}} \right).$$

A positive (negative) angle means that the biological series lags (leads) the climatic series. Means and variances of angles were computed using circular statistics^48^. For a specified frequency band (e.g., periods between 2 and 7 years), the distribution of phase angles was presented on a circular histogram (or wind rose), for time intervals within the cone of influence and with coherence *wc*_*n*_(*s*) > 0.5.

Further details on the wavelet transform methods used for this study, including significance testing, can be found in refs. ^36,46,47^. For applications to ecological studies see ref. ^49^. All analyses were performed in Matlab (2016b).

### Data availability

Further information about phenological data collection are available at http://stri.si.edu/sites/esp/tesp/plant_intro.htm, data are available from the authors.

Further information and unrestricted public access to meteorological data can be found at http://biogeodb.stri.si.edu/physical_monitoring/research/barrocolorado.

The wavelet coherence toolbox (WTC-R16) is available at http://www.glaciology.net/wavelet-coherence.

## Electronic supplementary material

Supplementary Information
