# Supplementary material for: Resource acquisition and reproductive strategies of tropical forest in response to the El Niño–Southern Oscillation
Source: Nat Commun. 2018 Mar 2;9:913. doi: 10.1038/s41467-018-03306-9 (PMC5834535; doi:10.1038/s41467-018-03306-9)
Supplement: Supplementary file 1 — Supplementary Information [file 41467_2018_3306_MOESM1_ESM.pdf]

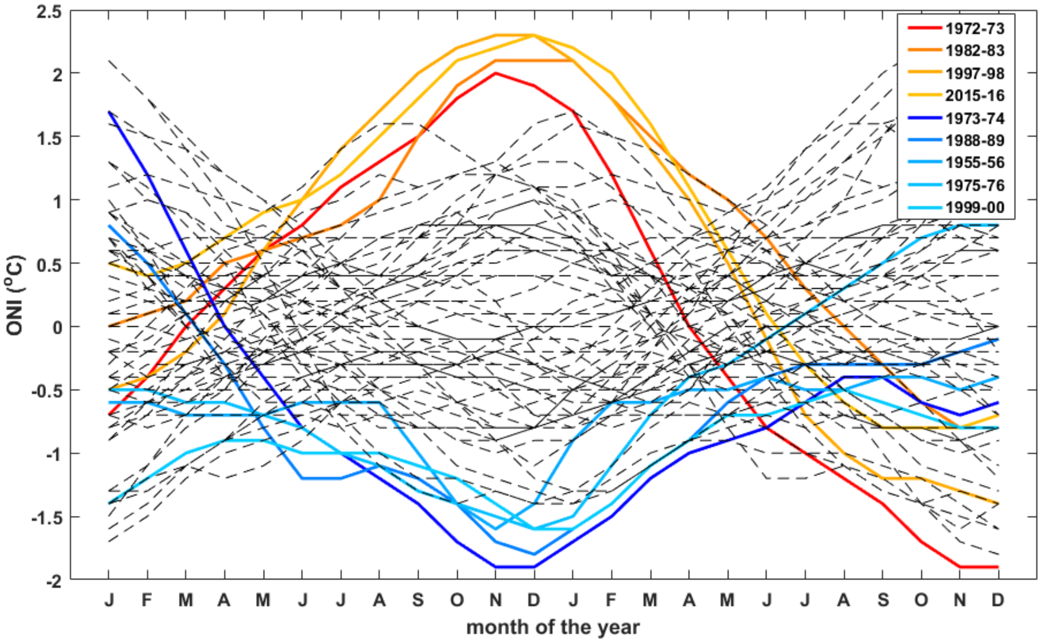

Supplementary Figure 1. Oceanic Nino Index plotted for two-year intervals in the period 1950-2016. Extreme years are indicated by red and blue lines denoting El Niño and La Niña events, respectively. Note that the peaks/troughs of these events are centered between October and January. Source: http://www.cpc.ncep.noaa.gov/products/analysis_monitoring/ensostuff/ensoyears.shtml.

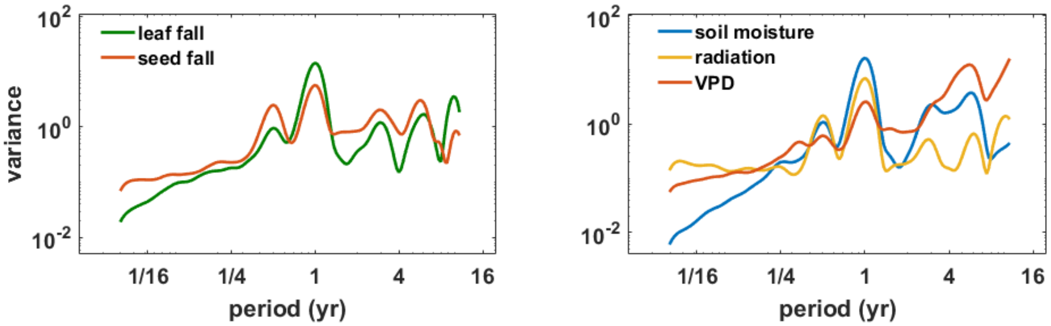

Supplementary Figure 2. Spectra of phenological series (left) and climatic series (right). All series were normalized to unit variance. The spectra show the contributions of each scale to the total variance in the series. The peaks at 1 year represent the seasonal cycle, which is the major contributor to the variance in every series. The sub-harmonic peak at 1/2 year is also related to the seasonal cycle and occurs because the oscillation is not a perfectly sinusoidal. Interannual scales (period >1 year) also contribute to the total variance, with two peaks in the ENSO cycle (2-7 years). At small periods, the variance decreases rapidly. There are notable differences in the spectra. Seed fall has relatively higher variability than leaf fall at ENSO scales. For the climatic variables, VPD exhibits higher variability in the interannual scales. The solar radiation spectrum is quite flat, except for the seasonal peak, resembling a white noise (equal variance at all scales).

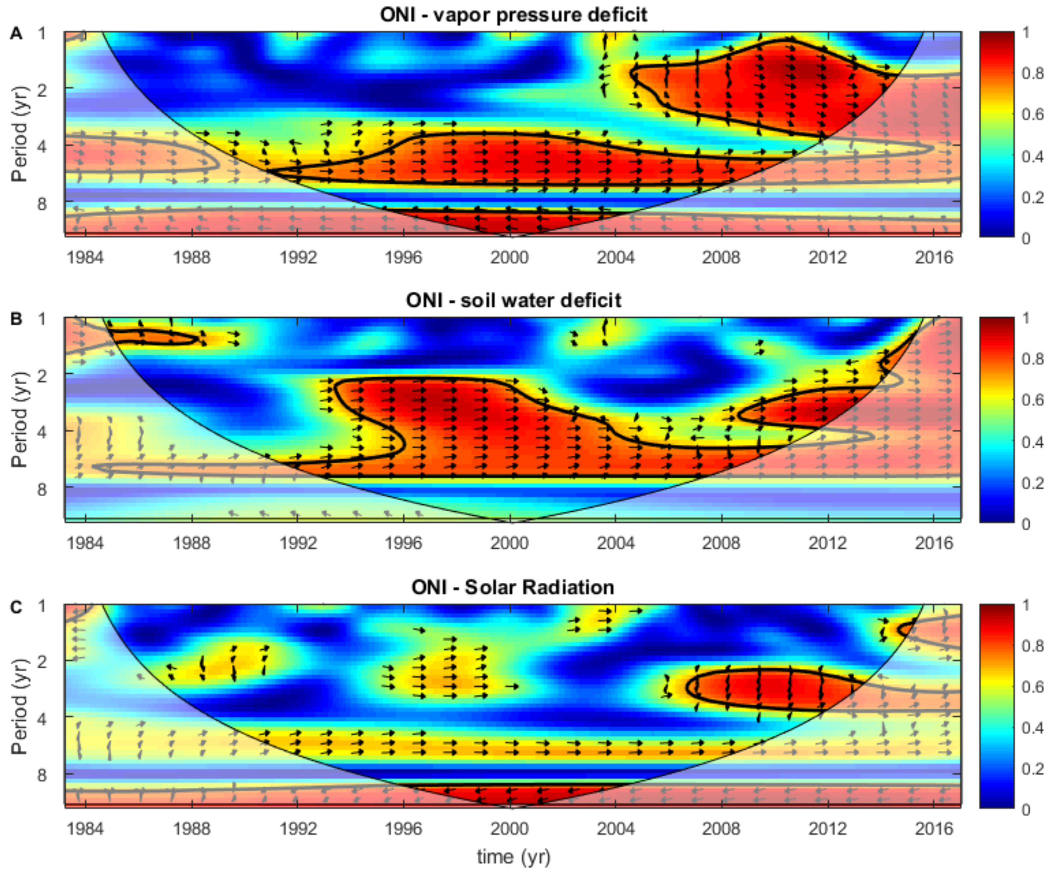

Supplementary Figure 3. Wavelet coherence analyses between ONI (Oceanic Nino Index) and local climate variables measured on Barro Colorado Island show the tendency towards warmer, drier, and sunnier conditions during El Niño periods. Solar radiation, although in phase with ENSO, exhibits relatively less correlation. Arrows indicate phase angle (the two series are in phase when the arrows point to the right). Red regions bounded by black contour lines indicate significant coherence ($\alpha < 0.05$).

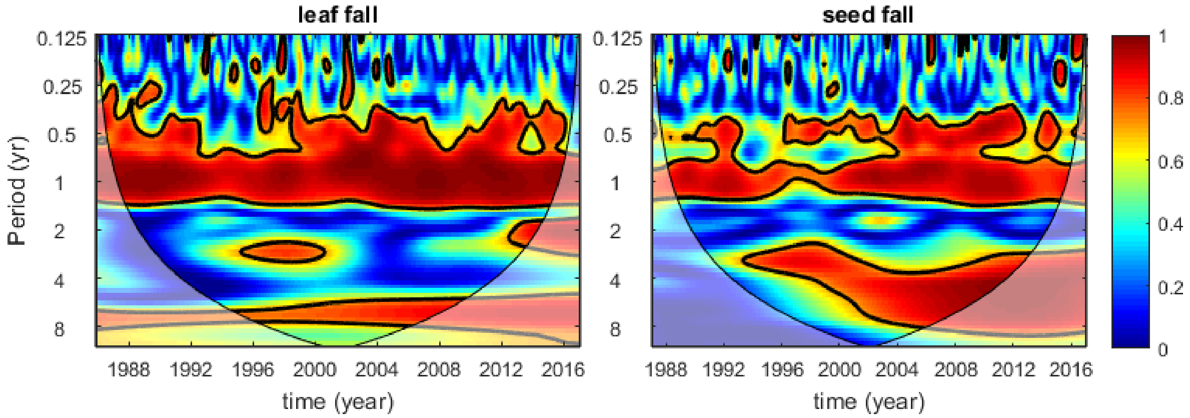

Supplementary Figure 4. Wavelet coherence analysis of seed fall and leaf fall with streamflow discharge, indicating strong connections at both seasonal (1 year) and ENSO cycles (2-7 year). Red regions bounded by black contour lines indicate significant coherence ($\alpha < 0.05$). Lighter shading indicates areas outside the cone of influence within which there are sufficient data to reliably test patterns at particular periods and times. Discharge was measured at the Lutz catchment area from the water level in a 120° V-notch weir (for further details visit http://biogeodb.stri.si.edu/physical_monitoring/research/barrocolorado).

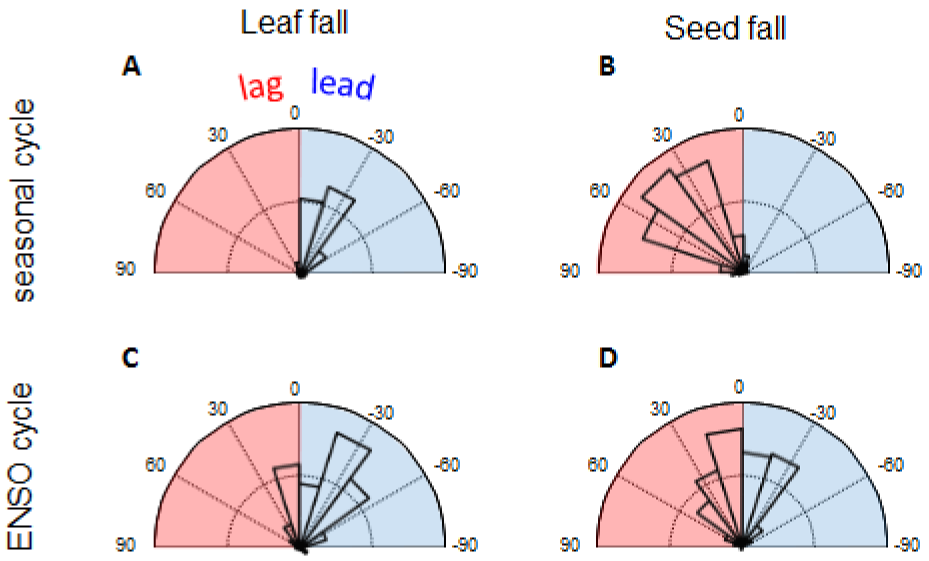

Supplementary Figure 5. Phase angle histograms for leaf and seed fall with solar radiation at seasonal cycles (12 months; panels A and B) and periods of 2-7 years, corresponding to ENSO cycles (panels C and D). Phase angles were calculated for areas inside the cone of influence and for coherence greater than 0.5 in figure 3. An angle of 30°corresponds to approximately one month for the seasonal cycle and 2-7 months for the ENSO cycle. Angles in the blue (red) shaded areas indicates that plant phenology leads (lags) climate.

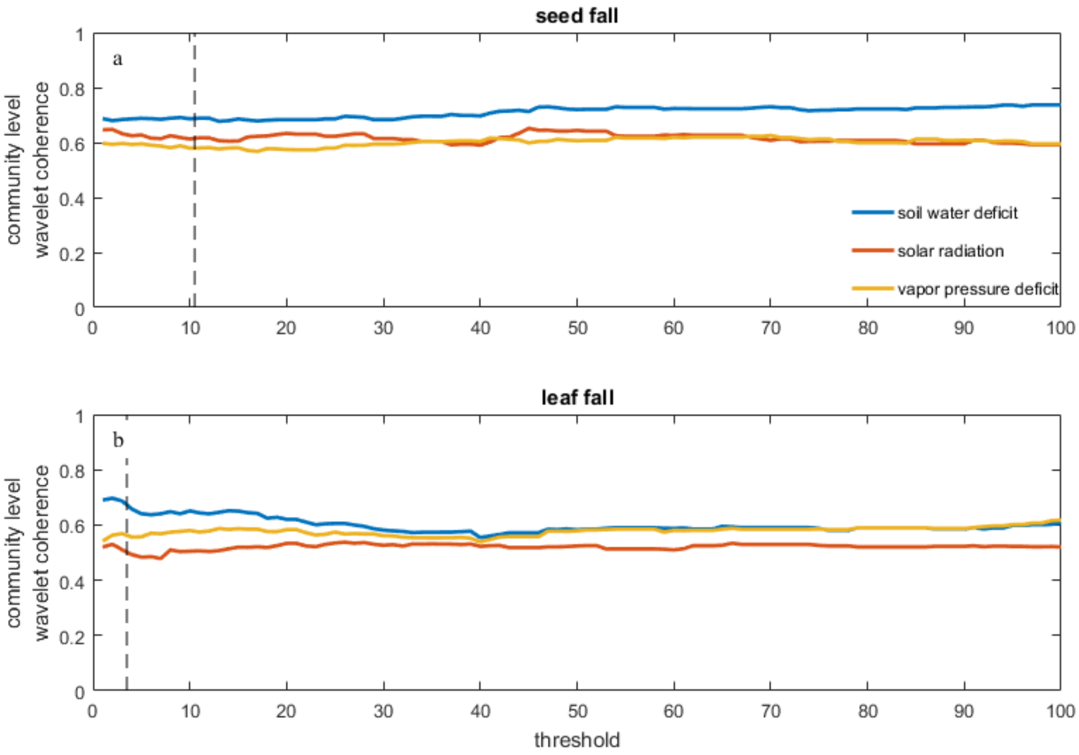

Supplementary Figure 6. Sensitivity of wavelet coherences in the ENSO period (2-7 years) to the minimum number of censuses for inclusion of species in the analyses for a) seed fall and climatic variables, and b) leaf fall and climatic variables. Vertical dashed lines indicate the thresholds used in this study (minimum of 11 and 4 censuses for seeds and leaves, respectively). The wavelet coherence is averaged inside the cone of influence (the region not biased by edge effects) for periods between 2 and 7 years, corresponding to the modes of ENSO. The analysis shows that the community-level coherence is fairly insensitive to this arbitrary threshold.
